# Defining the canal for ischial and pubic screws in cup revision surgery

**DOI:** 10.1007/s00264-022-05552-5

**Published:** 2022-08-22

**Authors:** Steffen Brodt, Vincent Boersch, Patrick Strube, Georgi Wassilew, Georg Matziolis

**Affiliations:** 1 German Center for Orthopaedics, Jena University Hospital, Campus Eisenberg, Klosterlausnitzer Straße 81, 07607 Eisenberg, Germany; 2 Clinic for Traumatology and Orthopedic Surgery, Klinikum Kassel, Kassel, Germany; 3 Clinic for Orthopedics and Orthopedic Surgery, University Medicine Greifswald, Greifswald, Germany

**Keywords:** THA, Revision, Acetabular screws, Screw positioning, Safe zone

## Abstract

**Purpose:**

When revising acetabular cups, it is often necessary to provide additional stabilisation with screws. In extensive defect situations, the placement of screws caudally in the ischium and/or pubis is biomechanically advantageous. Especially after multiple revision operations, the surgeon is confronted with a reduced bone stock and unclear or altered anatomy. In addition, screw placement caudally is associated with greater risk. Therefore, the present study aims to identify and define safe zones for the placement of caudal acetabular screws.

**Methods:**

Forty-three complete CT datasets were used for the evaluation. Sixty-three distinctive 3D points representing bone landmark of interests were defined. The coordinates of these points were then used to calculate all the parameters. For simplified visualisation and intra-operative reproducibility, an analogue clock was used, with 12 o’clock indicating cranial and 6 o’clock caudal.

**Results:**

A consistent accumulation was found at around 4.5 ± 0.3 hours for the ischium and 7.9 ± 0.3 hours for the pubic bone.

**Conclusions:**

The anatomy of the ischium and pubis is sufficiently constant to allow the positioning of screws in a standardised way. The interindividual variation is low — regardless of gender — so that the values determined can be used to position screws safely in the ischium and pubis. The values determined can provide the surgeon with additional orientation intra-operatively when placing caudal acetabular screws.

## Introduction

Based on the demographic development of the population and the number of implanted total hip replacements, a drastic increase in revision and re-revision operations on the hip joint is predicted [1, 2]. In the case of the first revision, the surgeon often still has sufficient bone stock available in which the respective defect situation can be managed with standard implants. In contrast, multiple revision operations or anatomical anomalies, such as hip dysplasia, often require so-called jumbo cups, reinforcement rings, modular cups with augments, flanged implants and patient-specific 3D-printed implants [3–7]. In addition to a stable anchorage in the remaining bone stock, the aim here is to reconstruct the centre of rotation as closely as possible to the original anatomy [8]. Classically, cranially placed screws are used. The corridor for cranial screw placement is sufficiently large and clearly defined [9, 10]. However, especially in the case of extensive defects or pelvic discontinuities such as Paprosky IIIA or IIIB defects, purely cranial fixation is not sufficient [11]. To ensure osseous integration of the revision implant, it is essential to ensure sufficient primary stability to avoid micromotion in the first weeks after surgery. The placement of inferior screws (ischial or pubic) can reduce the tilting moment and thus increase primary stability [12, 13]. Placement of the screws caudally is surgically more demanding than cranially. In order not to injure anatomically closely adjacent vascular and nerve structures, narrow osseous corridors are available for the insertion of screws into the pubic bone and ischium [14]. In the literature, as in practical application, a division into four quadrants has become established [9, 15, 16]. Here, the posterior superior quadrant for the ilium and the posterior inferior quadrant for the ischium are generally considered relatively safe for screw placement. In contrast, the anterior superior and anterior inferior quadrants should be avoided if possible, as important anatomical structures such as the external iliac artery and vein and the obturator artery, vein and nerve pass through them. However, a detailed description of the optimal screw placement is not available. It is well-known that there are distinctive anatomical differences between the male and female pelvis. While the male pelvis has been optimised for locomotion, the female pelvis has developed a maximum width for childbirth. Consequently, the male pelvis is narrower and taller, resulting in an acute subpubic angle. Conversely, the female pelvis is larger and wider, resulting in an oval pelvis inlet and an obtuse subpubic angle. Moreover, the female acetabula are wider apart and face more medially. It is likely that these differences in the anatomy of the bony pelvis will lead to differences when placing screws. Firstly, the angle at which the screws are fixed through the cup may differ and, secondly, the length of screws may vary due to the differences in pelvic size. In order to gain an insight into and potentially adapt to slight differences between the genders, these differences were to be investigated.

Currently, there are no detailed corridors or safe zones for placing screws into either the pubic bone or the ischium. The objective of the present study was to investigate this and establish safe zones, providing the orthopaedic surgeon with standard reference ranges for the angle at which screws should be placed and the length of screws to be used. The demand for simplicity is essential, because experience has shown that the acceptance and usage of such a system in everyday clinical practice is higher the less complex it is.

## Material and methods

### Data collection and collation

This study received the ethics approval by ethics committee from the Jena University Hospital (2020-1825-Daten) on June 15, 2020. The corresponding ethics approval number is 2020–1825-Daten.

QCT scans (BrightSpeed Series CT systems, GE Healthcare, Milwaukee, USA, slice thickness of 2 mm) from 43 patients from a previous study were used in this study [17]. The cohort investigated contained a Caucasian population with an even distribution of 22 men and 21 women. The average age was 62.0 years, ranging from 47 to 75, with a standard deviation of 6.7.

For data collection, the free open-source medical image viewer HOROS V3.3.6. was used.

The 3D raw data of each collected point was copied into Microsoft Excel® Version 16.46 (Redmond, Washington, USA). Using dedicated and validated trigonometric equations, the pelvic and acetabular plane and the ischial and pubic corridors as well as the relative position of those were calculated using Microsoft Excel®. All the values were calculated separately for both the left and the right hip. The calculated parameters are given in Tables 1 and 2.

**Table 1:** Parameters calculated in the study

Parameter	Definition
Sagittal angle of the ischial screw	The angle of the ischial screw in the sagittal plane with 0° pointing cranially
Clock position of the ischial screw	The position of the ischial screw described using a clock as a visual representation. The clock is oriented in the sagittal plane with 12 o’clock pointing cranially
Transverse angle of the ischial screw	The angle of the ischial screw in the transverse plane with regard to the acetabular inlet plane (this is an imaginary line that tangents both the anterior and posterior rim of the acetabulum)
Sagittal angle of the pubic screw	The angle of the pubic screw in the sagittal plane with 0° pointing cranially
Clock position of the pubic screw	The position of the pubic screw described using a clock as a visual representation. The clock is oriented in the sagittal plane with 12 o’clock pointing cranially
Transverse angle of the pubic screw	The angle of the pubic screw in the transverse plane with regard to the acetabular inlet plane (this is an imaginary line that tangents both the anterior and posterior rim of the acetabulum)
Length of ischial screw	The length of the ischial screw measured in millimetres
Length of pubic screw	The length of the pubic screw measured in millimetres

**Table 2:** Comparison of the dataset (mean values) for all male and female hips

	All	Female	Male	*p*-value
Clock position Ischium (o’clock)	4.5 ± 0.3	4.5 ± 0.2	4.5 ± 0.3	0.944
Ischial screw angle transverse plane (°)	35.6 ± 9.3	37.1 ± 8.7	34.1 ± 9.6	0.135
Clock position pubis (o’clock)	7.9 ± 0.3	7.9 ± 0.4	7.9 ± 0.3	0.537
Pubic screw angle transverse plane (°)	36.3 ± 9.5	37.4 ± 11.2	35.2 ± 7.5	0.296
Screw length ischium (mm)	52.3 ± 5.5	49.2 ± 4.1	55.1 ± 5.2	0.000
Screw length pubis (mm)	71.8 ± 4.8	72.4 ± 4.3	71.3 ± 5.3	0.259

**Fig. 1:**
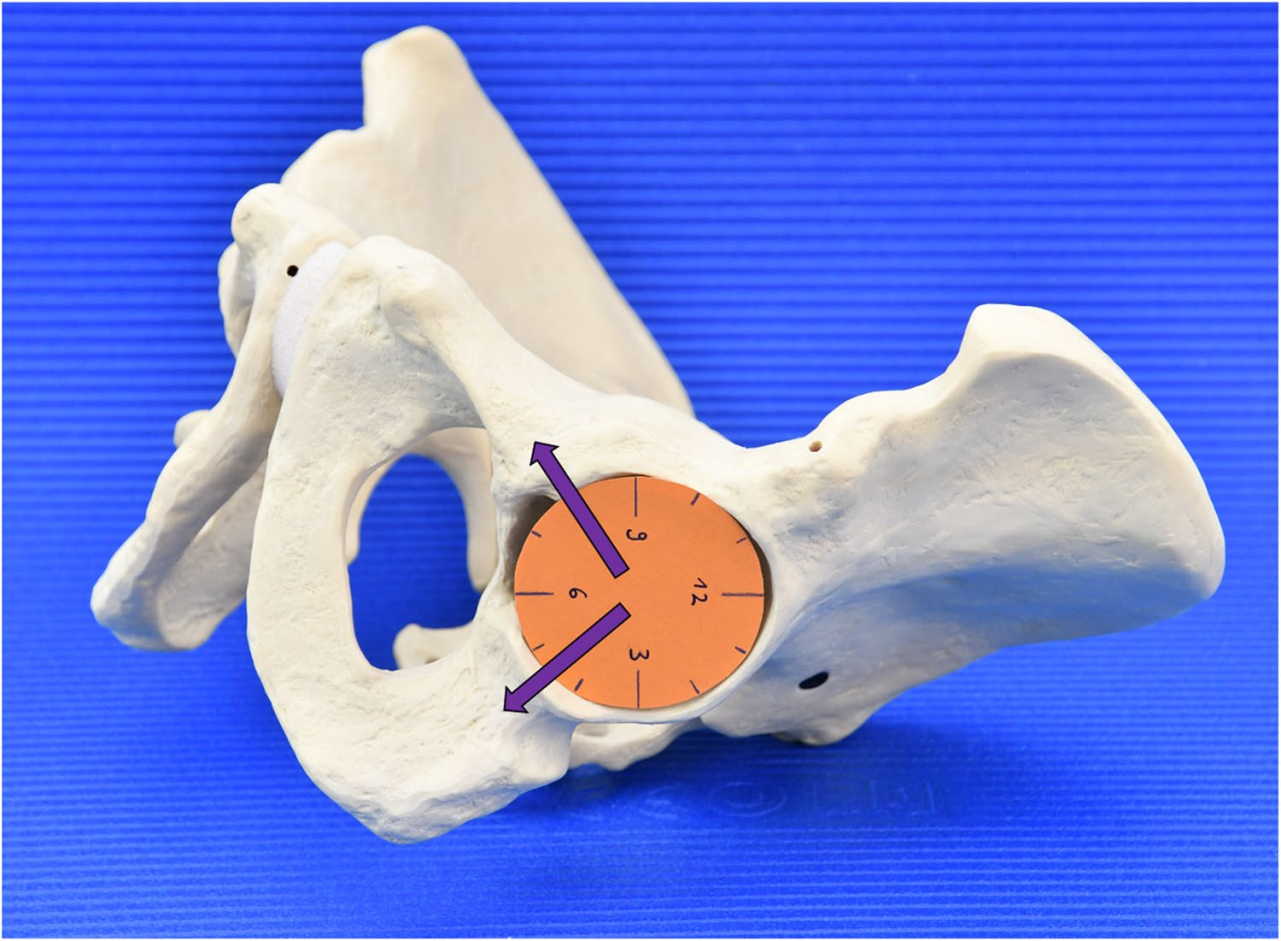
Clockwise orientation of the acetabulum. Arrows indicating clock position of the Ischium and Pubis

To simplify targeting the safe zones for the orthopaedic surgeon, we decided to use the analogy of a clock to represent the acetabulum (Fig. 1). The 12 is located cranially and the 6 caudally. Being able to identify these orienting landmarks will allow the surgeon to confidently use the clock system to place supporting screws in the ischial and pubic bones during revision surgery.

Prior to starting data collection, 63 distinctive 3D points representing bone landmarks of interest were defined. The coordinates of these points were then used to calculate all the parameters.

The first and second points were the right and left anterior superior iliac spine, respectively. Point number 3 was located at the symphysis. The next 30 points were all placed along the rim of right acetabulum. This process was then repeated for the left side.

**Fig. 2:**
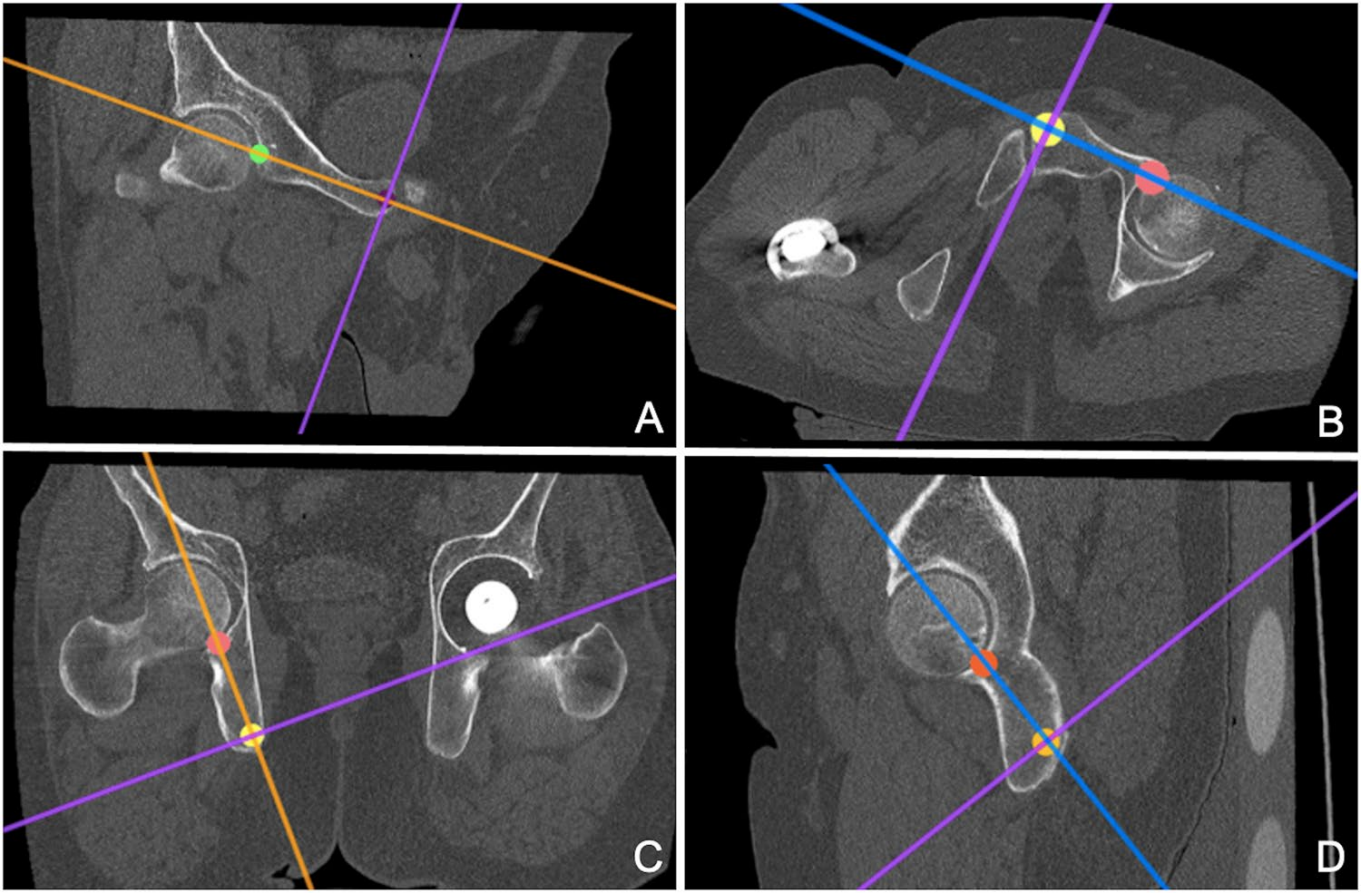
**A** Calculation of the pubic screw length in the coronal plane; **B** Calculation of the pubic screw length in thetransverse plane; **C** Calculation of the ischial screw length in the coronal plane; **D** Calculation of the ischial screwlength in the transverse plane

For this determination of optimal screw position, a different function within *HOROS* was used and the 3D view was selected. The CT scan was then positioned in such an orientation that the axis lines provided could be used as a reference for the path the screws would be taking. Next, one point was selected where the axis line intersected with the lateral border of the acetabular fossa, reflecting the point where the screw would enter into the direction of the pubic bone. Another point was selected at either the end of the pubic or ischial bone, representing the end point. Using the coordinates of these two points made it possible to calculate the maximum length a screw could have (Fig. 2A). In addition, the transverse plane of the CT scan was used to ensure that the axis line, which represents the path the screw would take, runs fully within the bone and does not intersect and injure any soft tissue (Fig. 2B).

Similarly, this process was then repeated for the ischium in both the coronal (Fig. 2C) and transverse plane (Fig. 2D). Both figures show that the axis line bisects the points placed within the CT scan. This process was then repeated for the left side. This generated four pairs of 3D coordinates, making it possible to calculate the distance between these points, corresponding to the maximum length of the screws.

## Results

Comparing the right with the left hip, no differences of clinical relevance were found, so that mean values were calculated for each patient.

Descriptive statistics of this dataset yielded the following results.

The table shows the distribution of the time equivalent of the ischial screw angle. There is a consistent accumulation at a mean value of 4.5 h for the ischium and a mean value of 7.9 h for the pubis. Both have a narrow spread, so that these values can be used in clinical routine. The data does contain one severe outlier. However, apart from this, the spread is very narrow, which leads to the conclusion that placing the ischial screw at 4.5 h is the optimal position with regard to the sagittal plane.

## Discussion

The main result of the present study is that the anatomy of the ischium and pubis is sufficiently constant to allow the positioning of screws in a standardised way. The interindividual variation is low — regardless of gender — so that the values determined can be used to position screws safely in the ischium and pubis. This not only increases the desired stability of the acetabular construct, but may also help to reduce the risk of vascular and nerve injury [9, 18].

The additional use of acetabular screws can increase the stability of a pressfit cup locally. Stranne et al. found that the stability of the cup is increased most when the screws are placed bicortically in the ilium, posterior column and the ischium [14]. Micromotion is mainly reduced locally in the area of the screw. Placing several screws directly next to each other in the ilium increases stability only slightly. In contrast, micromotion of the cup may even be increased inferiorly, in the area of the ischium [19, 20]. Consequently, the overall stability of the acetabulum is increased above all if the screws are not concentrated locally in the area of the acetabular dome but are inserted as far apart as possible in the area of the acetabular rim [21]. The insertion of acetabular screws, especially if more than one screw is used, can even change the position of the implant and thus theoretically increase the risk of dislocation [22]. In general, it is important to place the screws correctly, because eccentric or angulated screw placement can lead to a reduction in stability [23, 24].

The values determined in the present study are based on the evaluation of CT examinations. All patients evaluated here had normal bone anatomy. Therefore, caution should be exercised in transferring these results to cases of bone irregularities, such as those that can occur in dysplasia, after osteotomies or after fractures. This is especially true in the case of acetabular revision, where screws are used particularly frequently. Here, the surgeon often encounters qualitatively and quantitatively reduced bone stock with sometimes massive defects and an irregular, altered centre of rotation [25–27].

This fact also represents a limitation of the present study. The data presented can only be used with an original centre of rotation and acetabular entry plane, as the reference of the insertion angle of the screws is difficult to identify in the case of osseous defects of the acetabulum.

## Conclusion

Nevertheless, taking into account the above-mentioned limitations, an orientation towards the values suggested here is helpful in determining the starting point and primary direction of drilling. This can help the inexperienced surgeon or if an intraoperative x-ray or image intensifier is not available. We still recommend that final drilling of caudal screws be carried out under image intensifier, visual and palpatory control in order to avoid misplacements. In centres where revision surgery in hip arthroplasty is performed, intraoperative imaging should be at least available.

## Data Availability

From the author.

## References

[1] Kurtz S, Ong K, Lau E (2007). Projections of primary and revision hip and knee arthroplasty in the United States from 2005 to 2030. J Bone Joint Surg Am.

[2] Patel A, Pavlou G, Mújica-Mota RE, Toms AD (2015). The epidemiology of revision total knee and hip arthroplasty in England and Wales: a comparative analysis with projections for the United States. A study using the National Joint Registry dataset. Bone Joint J.

[3] Durand-Hill M, Henckel J, Di Laura A, Hart AJ (2020). Can custom 3D printed implants successfully reconstruct massive acetabular defects? A 3D-CT assessment. J Orthop Res.

[4] Walter SG, Randau TM, Gravius N (2020). Monoflanged custom-made acetabular components promote biomechanical restoration of severe acetabular bone defects by metallic defect reconstruction. J Arthroplasty.

[5] Zhou B, Zhou Y, Yang D (2018). The utilization of metal augments allows better biomechanical reconstruction of the hip in revision total hip arthroplasty with severe acetabular defects: a comparative study. J Arthroplasty.

[6] Dearborn JT, Harris WH (2000). Acetabular revision arthroplasty using so-called jumbo cementless components: an average 7-year follow-up study. J Arthroplasty.

[7] Berry DJ, Müller ME (1992). Revision arthroplasty using an anti-protrusio cage for massive acetabular bone deficiency. J Bone Joint Surg Br.

[8] Khlopas A, Chughtai M, Elmallah RK (2018). Novel acetabular cup for revision THA improves hip center of rotation: a radiographic evaluation. Clin Orthop Relat Res.

[9] Wasielewski RC, Cooperstein LA, Kruger MP, Rubash HE (1990). Acetabular anatomy and the transacetabular fixation of screws in total hip arthroplasty. J Bone Joint Surg Am.

[10] Faizan A, Black BJ, Fay BD (2016). Comparison of head center position and screw fixation options between a jumbo cup and an offset center of rotation cup in revision total hip arthroplasty: a computer simulation study. J Arthroplasty.

[11] Paprosky WG, Perona PG, Lawrence JM (1994). Acetabular defect classification and surgical reconstruction in revision arthroplasty. A 6-year follow-up evaluation. J Arthroplasty.

[12] Solomon LB, Abrahams JM, Callary SA, Howie DW (2018). The stability of the porous tantalum components used in revision THA to treat severe acetabular defects: a radiostereometric analysis study. J Bone Joint Surg Am.

[13] Meneghini RM, Stultz AD, Watson JS (2010). Does ischial screw fixation improve mechanical stability in revision total hip arthroplasty?. J Arthroplasty.

[14] Stranne SK, Callaghan JJ, Elder SH (1991). Screw-augmented fixation of acetabular components. A mechanical model to determine optimal screw placement. J Arthroplasty.

[15] Meldrum R, Johansen RL (2001). Safe screw placement in acetabular revision surgery. J Arthroplasty.

[16] Dietze S, Perka C, Baecker H (2014). Blood vessel and nerve damage in total hip arthroplasty. Orthopade.

[17] Graul I, Strube P, Vogt S (2022). Does total hip arthroplasty influence the development and localization of sacral insufficiency fractures?. J Bone Joint Surg Am.

[18] Singh NK, Rai SK, Rastogi A (2017). Possible vascular injury due to screw eccentricity in minimally invasive total hip arthroplasty. Indian J Orthop.

[19] Hsu J-T, Chang C-H, Huang H-L (2007). The number of screws, bone quality, and friction coefficient affect acetabular cup stability. Med Eng Phys.

[20] Won CH, Hearn TC, Tile M (1995). Micromotion of cementless hemispherical acetabular components. Does press-fit need adjunctive screw fixation?. J Bone Joint Surg Br.

[21] Hsu J-T, Lai K-A, Chen Q (2006). The relation between micromotion and screw fixation in acetabular cup. Comput Methods Programs Biomed.

[22] Fujishiro T, Hayashi S, Kanzaki N (2014). Effect of screw fixation on acetabular component alignment change in total hip arthroplasty. Int Orthop (SICOT).

[23] Hsu J-T, Chang C-H, An K-N (2007). Effects of screw eccentricity on the initial stability of the acetabular cup. Int Orthop SICO.

[24] Hsu J-T, Lin D-J (2010). Effects of screw eccentricity on the initial stability of the acetabular cup in artificial foam bone of different qualities. Artif Organs.

[25] Dennis DA (2003). Management of massive acetabular defects in revision total hip arthroplasty. J Arthroplasty.

[26] Sculco PK, Wright T, Malahias M-A (2022). The diagnosis and treatment of acetabular bone loss in revision hip arthroplasty: an international consensus symposium. HSS J.

[27] Wasielewski RC, Galat DD, Sheridan KC, Rubash HE (2005) Acetabular anatomy and transacetabular screw fixation at the high hip center: clinical orthopaedics and related research NA;171–176. 10.1097/01.blo.0000165855.76244.53 10.1097/01.blo.0000165855.76244.53 16131887

